# Improved benchmark-multiplier method to estimate the prevalence of ever-injecting drug use in Belgium, 2000–10

**DOI:** 10.1186/0778-7367-71-10

**Published:** 2013-05-03

**Authors:** Kaatje Bollaerts, Marc Aerts, Andre Sasse

**Affiliations:** 1 OD Public Health and Surveillance, Scientific Institute Public Health, Rue Juliette Wytsmanstraat 14, Brussels B-1050, Belgium; 2 Interuniversity Institute for Biostatistics and Statistical Bioinformatics, Hasselt University & Catholic University, Diepenbeek, Leuven, Belgium

**Keywords:** Population size estimation, Ever injecting drug use, Benchmark-multiplier method, HIV/AIDS register, Imputation by Chained Equations, Stochastic mortality modelling

## Abstract

**Background:**

Accurate estimates of the size of the drug-using populations are essential for evidence-based policy making. However, drug users form a ‘hidden’ population, necessitating the use of indirect methods to estimate population sizes.

**Methods:**

The benchmark-multiplier method was applied to estimate the population size of ever injecting drug users (ever-IDUs), aged 18–64 years, in Belgium using data from the national HIV/AIDS register and from a sero-behavioral study among injecting drug users. However, missing risk factor information and absence of follow-up of the HIV^+^/AIDS^–^ cases, limits the usefulness of the Belgian HIV/AIDS register as benchmark. To overcome these limitations, statistical corrections were required. In particular, Imputation by Chained Equations was used to correct for the missing risk factor information whereas stochastic mortality modelling was applied to account for the mortality among the HIV^+^/AIDS^–^ cases. Monte Carlo simulation was used to obtain confidence intervals, properly reflecting the uncertainty due to random error as well as the uncertainty associated with the two statistical corrections mentioned above.

**Results:**

In 2010, the prevalence (/1000) of ever-IDUs was estimated to be 3.5 with 95% confidence interval [2.5;4.8]. No significant time trends were observed for the period 2000–2010.

**Conclusions:**

To be able to estimate the ever-IDU population size using the Belgian HIV/AIDS register as benchmark, statistical corrections were required without which seriously biased estimates would result. By developing the improved methodology, Belgium is again able to provide ever-IDU population estimates, which are essential to assess the coverage of treatment and to forecast health care needs and costs.

## Background

Accurate estimates of the size of the drug-using population are indispensable to govern and evaluate drug policy. They are particularly important to forecast health care needs and costs and to assess the coverage of treatment and harm reduction measures e.g. [1,2]. However, drug users form a `hidden’ population, hiding their membership because it involves illegal and stigmatized behaviour. As a result, `hidden’ populations lack sampling frames and classical epidemiological methods (e.g. population-based surveys) fail. Therefore, indirect estimation methods are to be used. Generally, three types of indirect estimation methods are distinguished to estimate the size of drug using populations: capture-recapture (CR), benchmark-multiplier (BM) and multivariate indicator (MI) methods [3-5]. An application of the BM method is presented in this paper.

The BM method is a very intuitive method, relatively easy to apply and hence, frequently used [6]. The BM method combines (an estimate of) the size of the known part of the target population, the benchmark, with an estimate of the proportion of individuals from the target population belonging to the benchmark. The reciprocal of the proportion is called the multiplier. The size of the target population is then estimated as the product of the benchmark and the multiplier. Common sources used as benchmark are mortality registers, HIV/AIDS registers, drug treatment or police data e.g. [7,8]. During the beginning of the 1970s, the mortality multiplier method was already used [9]. Multiplier methods e.g. [10,11] have produced estimates consistent with estimates produced by CR methods, which have been put forward as a preferred methodology [6]. The key assumptions underlying the BM method are that the benchmark should be exhaustive, the sample used to estimate the multiplier should be representative of the target population and the case definition used for the benchmark should exactly match the one used for the multiplier, including the time window [12].

In this paper, the benchmark-multiplier (BM) method was applied to estimate the prevalence of ever injecting drug users (ever-IDUs) within the population aged 15–64 years in Belgium, 2000–10, using the HIV/AIDS register as benchmark. In Belgium, HIV-screening is widely used and all sera of which the screening test result was positive, are submitted for confirmation to one of the seven AIDS Reference Laboratories (ARLs) in Belgium. The registration results of all ARLs are then send to the national HIV/AIDS register. As the seven ARLs are the only laboratories subsidized for performing HIV confirmation tests, the Belgian HIV/AIDS register is deemed to be exhaustive and hence, constitutes a potential suitable benchmark.

For Belgium, the latest estimate of the ever-IDU prevalence date from 1995 and was obtained adopting the BM approach using the HIV/AIDS register as benchmark [13]. However, the Belgium HIV/AIDS register suffers from missing risk factor information. Particularly, of all registered cases in 2000–10, 28.6% were reported without information on probably mode of HIV transmission (e.g. sexual contact, blood transfusion, injecting drug use). Simply discarding these cases from analysis would lead to an underestimation of the IDU prevalence. Simply extrapolating the risk factor fractions of the subpopulation for which the risk factors are known to the subpopulation for which they are unknown, would lead to overconfident results and would only lead to unbiased results under very strict assumptions (i.e. the Missing Completely ad Random assumption). In addition, the national HIV/AIDS register lacks follow-up of the non-AIDS cases, implying absence of information on the vital status of the HIV^+^/AIDS^–^ cases. Not accounting for the mortality among these cases would result in an overestimation of the number of alive seropositive IDUs, with the bias increasing as the time since the onset of the HIV-epidemic (mid-eighties) increases. The methodology proposed in [13] used a simple extrapolation method to account for the missing risk factor information and failed to account for the mortality among HIV^+^/AIDS^–^ cases. Therefore, the current paper presents an improved application of HIV/AIDS BM method, using up-to-date statistical methods to correct for the missing risk factor information and lacking follow-up of the HIV^+^/AIDS^–^ cases.

## Methods

### Benchmark: national HIV/AIDS register

During 2000–10, an average of 56 screening tests per 1000 inhabitants per year were performed in Belgium, excluding tests related to blood donations (National Institute for Sickness and Invalidity Insurance). The confirmation tests are performed by one of the seven ARLs in Belgium, being the only laboratories subsidized for performing these tests. The registration results of the seven ARLs are validated for duplicate recording and are all included in the national HIV/AIDS register, being hosted by the Scientific Institute of Public Health, Brussels (WIV-ISP). Since its foundation in 1985–86, the Belgian HIV/AIDS register is deemed to be exhaustive.

For each confirmed HIV-positive test, a standardized form is sent to the patient’s clinician to collect additional information on nationality, residence, sexual orientation, probable mode of HIV transmission and CD4 count at time of HIV diagnosis. The response categories for the self-reported probable mode of HIV transmission are homo- and heterosexual transmission, transmission through blood transfusion, transmission through injecting drug use and mother-to-child transmission. A person can indicate multiple modes of transmission. Unfortunately, the standardized forms are not always fully completed returned to the WIV-ISP, resulting in missing risk factor information.

Follow-up is conducted for patients who developed AIDS with each year data being collected on last consultation and possible death. The variables available for the AIDS cases are year at AIDS-diagnosis, year at death and year at lost to follow-up, which are only completed upon occurrence of the event and hence, are time dependent. The HIV^+^/AIDS^–^ cases are not subject to follow-up.

### Multiplier: sero-behavioral prevalence study

In Belgium, a sero-behavioral study among drug users in contact with drug treatment facilities or imprisoned was carried out in 2004–05 [14]. In total, 1005 drug users in treatment and 117 incarcerated drug users (15–40 years) enrolled at 65 different drug treatment facilities and 15 different prisons geographically dispersed over Belgium, respectively, participated to the study. Of the drug users in treatment and in prison, 57% (n = 573) and 68% (n = 80) declared to have injected drugs at least once during their life. Intravenous blood samples were taken to determine the HIV- as well as the Hepatitis B and -C status of the participants. The HIV-seroprevalence among IDUs in treatment and in prison was estimated to be 2.8% (95% Wald CI: [1.5;4.2]) and 5% (95% Wald CI:[0.2;9.9]), respectively. These prevalences were not significantly different (p-value = 0.30), yielding an overall estimated prevalence of 3.1% (95% Wald CI: [1.8;4.8]).

In addition to serological studies, the HIV prevalence among IDUs in Belgium can be obtained from routine diagnostic testing, of which the results are yearly available. This allows the investigation of time trends. However, a concern regarding the (geographical) representativeness of the Belgian data exists. In line with (Western) European trends [15], no significant time trends in HIV prevalences among IDUs were observed during the last 10 years in Belgium based on the results from routine diagnostic testing [16]. Therefore, the HIV prevalence from the sero-behavioral study was assumed to apply for the entire period 2000–10.

### Statistical methods

An estimate of the prevalence of ever-IDUs was obtained by means of the BM method. For the current application, the benchmark *N*_*x*_ was the number of alive seropositive ever-IDUs (aged 18–64 years) in Belgium for a given year. The benchmark *N*_*x*_ was obtained from the national HIV/AIDS register after correcting for the missing risk factor information using Imputation by Chained Equations [17-19] and after correcting for the lacking information on the vital status of the HIV^+^/AIDS^–^ cases using stochastic mortality modelling [20]. The multiplier *π* was then the reciprocal of the HIV-prevalence among the ever-IDUs, for which an estimate could be obtained from the sero-behavioral study by Plasschaert et al. [14]. A 3-step Monte Carlo simulation model was built to generate (interval) estimates of the prevalence of ever-IDUs. A schematic overview of the 3-step simulation model is provided in Table 1. The different steps within the simulation model are described in more detail below. This model was run K = 1000 times, yielding K = 1000 different estimates of the ever-IDU prevalence, based on which the 95% percentile Monte Carlo confidence intervals (95% MC CIs) were calculated.

**Table 1:** **Schematic overview of the 3-step Monte Carlo simulation model to estimate ever IDU prevalences**^**Ɨ**^

**STEP 1:**	**Imputation by Chained Equations: missing risk factor information**
	Missing risk factor information was imputed using Imputation by Chained Equations. The imputation model contains the variables: injecting drug use, sex, nationality, year at registration and age at registration. The imputation results in one complete dataset **X**_**k**_, containing original and imputed values.
**STEP 2:**	**Stochastic Mortality Modeling: lacking follow-up of the HIV**^**+**^**/AIDS**^**–**^**cases**
	For a complete dataset *k,* the number of registered HIV-cases for whom injecting drug use was the most probable route of transmission and who were alive at time *t* is calculated as ${\hat{N}}_{x}^{k}\left(t\right)=\sum_{i=1}^{n_{t}}\left(I_{i}^{k}\left|X_{3\;i}^{k}=1\right)\right)\left(t\right)\;\mathrm{with}\;I_{i}^{k}=\left\{\begin{array}{l} 0,\;T_{d\;i}<t\;\mathrm{or}\;T_{l\;i}<t\;\\ 1,\;T_{a\;i}\;\geq \;t\;\mathrm{or}\;T_{d\;i}\;\geq \;t\;\mathrm{or}\;T_{l\;i}\;\geq \;t\;\\ S_{i}\sim \mathrm{bern}\;\left({\left(1-p_{d}\right)}^{r_{i}}\right),\;\mathrm{otherwise},\; \end{array}\right)$ where *I*_*i*_ indicates the ‘vital’ status with *I*_*i*_ = 1 if person *i* is still alive and living in Belgium and *I*_*i*_ = 0 otherwise, where *r*_*i*_ is the number of years since HIV registration or *r*_*i*_ = *t* − *t*_*hi*_ and where *p*_*d*_ is the annual non-AIDS mortality rate among seropositive IDUs with *p*_*d*_ ~ betapert*(0.58*%*, 1.08*%*, 1.58*%*).
**STEP 3:**	**Benchmark-multiplier method: population size estimation**
	The number of ever-injecting drug users being alive at time *t* is given by ${\hat{N}}_{y}^{k}\left(t\right)={\hat{p}}_{\mathrm{HIV}}^{-1}{\hat{N}}_{x}^{k}\left(t\right)-n^{-1}{\hat{N}}_{x}^{k}\left(t\right)\left(1-{\hat{p}}_{\mathrm{HIV}}\right){\hat{p}}_{\mathrm{HIV}}^{-1},$ with ${\hat{N}}_{x}^{k}\left(t\right)$ obtained from step 2, ${\hat{p}}_{\mathrm{HIV}}\sim \mathrm{beta}\left(21,620\right)$ and *n* = 639.

Ɨ The model was run K = 1000 times.

* The betapert distribution is mainly used to model expert estimates and requires a minimum, most likely and maximum value.

Step 1: To correct for the missing risk factor information, *Imputation by Chained Equations* (ICE) was used. ICE is an iterative technique that starts by filling in the missing values in a simple way, e.g. by using mean imputation [17-19]. Then, each variable included in the imputation model *X*_*i*_, *i* = 1, 2,…*q*, is imputed in turn by the predictions of the regression model regressing the observed values of *X*_*i*_ on the observed and imputed values of the remaining variables within the imputation model. The iteration through all *q* variables constitutes one cycle. After a sufficient number of cycles (typically 10 cycles), the final imputations are retained, resulting in one ‘complete’ dataset. There are several software packages available providing (M)ICE procedures, i.e. STATA [21], S-PLUS or R [22]. For the current application, ICE as implemented in STATA was used to account for the missing risk factor information. In particular, the imputation model included all (auxiliary) variables that had a statistically significant association with the variable indicating whether injecting drug use was the most probable route of transmission; being sex, nationality, year at registration and age at registration. For every run *k* (*k* = 1,2,…K = 1000), one ‘complete’ dataset was generated.

Step 2: *Binomial stochastic mortality modelling* was applied to correct for the lacking information on the vital status of the HIV^+^/AIDS^–^ cases. More precisely, the vital status of a HIV^+^/AIDS^–^ case *i* was simulated using a binomial process having survival probability $p_{\;i{\;}_{}}={\left(1-p_{d}\right)}^{r_{i\;}}$ with $r_{i}$ being the number of years since HIV registration, implying lower survival probabilities when the time since HIV-registration increases. The non-AIDS crude mortality rate among IDUs (*p*_*d*_) was assumed to follow a betapert distribution [23] with minimum, most likely and maximum values of 0.58%, 1.08% and 1.58% per annum, respectively. These non-AIDS crude mortality rates were calculated by Degenhardt et al. [24] based on 44 cohort-studies. The vital status of the AIDS cases at time *t* was deduced from the AIDS-specific variables year at AIDS-diagnosis (T_a_), year at death (T_d_), and year at lost to follow-up (T_l_). In particular, an AIDS case was indicated to be alive at time *t* if any information was recorded after time *t* (i.e. if T_a_ ≥ *t* or T_d_ ≥ *t* or T_l_ ≥ *t*). On the other hand, an AIDS case was indicated to be death at time *t* if the person died before time *t* or was lost to follow-up. In the latter case, the person is most likely to be death or to have left the country.

Step 3: The *Benchmark Multiplier method* was obtained to estimate the size *N*_*y*_ of the partly ‘hidden’ population or the target population as

(1) $${\hat{N}}_{y}=\frac{{\hat{N}}_{x}}{\hat{\pi }},$$

having estimated benchmark ${\hat{N}}_{x}$ and estimated multiplier ${\hat{\pi }}^{-1}$. However, even if ${\hat{N}}_{x}$ and $\hat{\pi }$ are unbiased estimators, ${\hat{N}}_{y}$ is a biased estimator of *N*_*y*_ because of its non-linearity with respect to $\hat{\pi }$. Therefore, the bias-corrected estimator as proposed in (Bollaerts K, Sasse A, Aerts M: The benchmark-multiplier method: bias and new bias-corrected estimator. *In preparation*) was used or

(2) $${\hat{N}}_{y}^{\mathrm{BC}}=\frac{{\hat{N}}_{x}}{\hat{\pi }}-\frac{1}{n}{\hat{N}}_{x}\frac{\left(1-\hat{\pi }\right)}{\hat{\pi }},$$

with *n* being the size of sample used to estimate *π .* Observe that the sample size *n* is the only additional piece of information needed to construct the bias-corrected estimator as compared to the traditional estimator. Then, the estimate of the benchmark ${\hat{N}}_{x}$ was obtained from step 1 and 2 whereas the estimate $\hat{\pi }$ and the corresponding sample size *n* were derived from the sero-behavioral prevalence study [14]. As the study indicated that x = 20 out of the *n* = 639 ever-IDUs were seropositive, $\hat{\pi }$ was assumed to follow a beta distribution with $\hat{\pi }\sim \mathrm{beta}\left(x+1,n-x+1\right)$ or $\hat{\pi }\sim \mathrm{beta}\left(21,620\right)$[25].

## Results

After having corrected for missing risk factor information, a total of 845 HIV-cases (aged 18-64yrs) with the HIV-infection probably related to injecting drug use has been identified in Belgium up to the year 2000. This number increased to 1113 in 2010. Correcting for HIV^+^/AIDS^–^ mortality, these numbers dropped to 642 and 809 alive HIV^+^ ever-IDUs in 2000 and 2010, respectively. In 2010, the total number of ever-IDUs (aged 18-64yrs) was estimated to be 24664 (95% MC CI: [17565;34403]) with the corresponding prevalence (/1000) being 3.5 (95% MC CI: [2.5;4.8]). No significant time trends in the number of ever-IDUs were observed (Table 2).

**Table 2:** Results of the Monte Carlo (MC) simulation study estimating the prevalence of ever Injecting Drug Use (IDU), Belgium, 2000-10

	**Number of seropositive IDUs**		**Number of alive seropositive IDUs**		**Number of alive IDUs**		**Prevalence (/1000)**	
**Year**	Est^*^.	**95% MI CI**^**Ɨ**^	Est^*^.	**95% MI CI**^**Ɨ**^	Est^*^.	**95% MI CI**^**Ɨ**^	Est^*^.	**95% MI CI**^**Ɨ**^
2000	845	[814;877]	642	[612;673]	19497	[13540;27010]	2,9	[2.0;4.0]
2001	876	[848;906]	659	[630;690]	19989	[14147;27663]	3	[2.1;4.1]
2002	909	[879;940]	682	[654;713]	20721	[14724;28166]	3,1	[2.2;4.2]
2003	961	[932;993]	723	[692;755]	22191	[15927;30659]	3,3	[2.3;4.5]
2004	995	[964;1028]	744	[712;775]	22861	[16220;31947]	3,4	[2.4;4.7]
2005	1009	[978;1042]	749	[719;781]	22653	[15743;31955]	3,3	[2.3;4.7]
2006	1031	[997;1063]	758	[726;790]	23178	[16362;31976]	3,4	[2.4;4.6]
2007	1061	[1029;1095]	779	[747;812]	23704	[16583;32937]	3,4	[2.4;4.7]
2008	1080	[1049;1112]	792	[761;826]	24026	[16844;32958]	3,4	[2.4;4.7]
2009	1094	[1062;1127]	798	[766;831]	24358	[17013;33951]	3,4	[2.4;4.8]
2010	1113	[1079;1147]	809	[775;842]	24664	[17565;34403]	3,5	[2.5;4.8]

^*^ Est.: mean estimate; ^Ɨ^ 95% MC CI: 95% Monte Carlo Confidence Intervals.

## Discussion

To obtain recent estimates of the ever-IDU prevalence in Belgium, an improved HIV multiplier method was applied. The improved methodology allows using the Belgian HIV/AIDS register as benchmark while accounting for missing risk factor information (through Imputation by Chained Equations - ICE) and lacking follow-up of the HIV^+^/AIDS^–^ cases (through stochastic mortality modelling). The statistical corrections were implemented in the best possible way. Indeed, ICE is a very flexible imputation approach, that can deal with several incomplete variables, does not require specific missing data patterns and operates under the less stringent assumption that the probability of the missing values depends only on the observed values and not on unobserved values (the so-called Missing at Random assumption, [26]) [17-19]. The stochastic mortality model accounts for the uncertainty in the mortality rate and the Monte Carlo simulation model properly acknowledges the uncertainty associated with the statistical corrections mentioned above.

In 2010, the prevalence (per 1000 inhabitants, 15–64 years) of ever injecting drug use in Belgium was estimated to be 3.5 (95% CI: 2.5-4.8), with no significant time trends being observed for the period 2000–10. Similar findings were reported by the other European countries providing time trends of the national prevalence of ever injecting drug use, i.e. Norway, Cyprus and the Czech republic [15]. This absence of a time trend is seemingly in contrast with recent findings from the EMCDDA treatment demand indicator, suggesting a decline in recent-onset heroin users and heroin injectors in the Western European countries [27]. However, it need to be stressed that the prevalence of ever injecting drug use is less sensitive to recent trends in drug use.

The above mentioned results should also be regarded in the light of the assumptions underlying the BM method. Particularly, the BM estimates rely on the conditions that the benchmark is complete and that the multiplier is estimated based on a representative sample of the target population. As benchmark of the current analysis, the national HIV/AIDS register was used. Since its foundation, the Belgian HIV/AIDS register is deemed to be exhaustive as it includes the results of all Belgian laboratories subsidised for performing HIV confirmation tests. As not only AIDS cases but also HIV cases are registered, the Belgian HIV/AIDS register has the additional advantage that back-calculation methods are not needed to obtain the HIV prevalence. The multiplier was obtained from a national sero-prevalence study including 653 IDUs [14]. Although this is the largest and most recent Belgian sero-prevalence study, it did not include IDUs outside treatment/prison, for whom currently no information is available. Despite the fact that low threshold drugs services are common in Belgium, the possibility of bias resulting from a multiplier derived from a sample that is not fully representative of the target population cannot be ruled out.

To summarize, the current paper provides a thorough application of the BM method. Nevertheless, the use of such indirect methods inherently relies on empirically non-verifiable (but reasonable) assumptions, such as the representativeness of the multiplier. Therefore, the current estimate of the ever IDU prevalence should be complemented with estimates based on other data sources (e.g. substitution treatment register) and/or using other methods (e.g.capture-recapture, multivariate indicator method) (e.g. [28]). For now, it is (reasonably) assumed that the HIV prevalence among IDUs remained stable during the last ten years. However, new prevalence estimates are needed for future applications of the BM method based on the Belgian HIV/AIDS register. Finally, this paper presents estimates of the ever IDU prevalence, which are important to assess future health care needs regarding e.g. Hepatitis C treatment. However, when interest is in assessing the coverage adequacy of needle exchange programmes (NEPs) or opioid substitution treatment (OST), national estimates of the current IDU prevalence are required. The latter prevalence (per 1000 inhabitants, 15–64 years) can be roughly estimated as 1.4 (95% CI: 1.02-1.97) by multiplying the ever-IDU prevalence with the ratio between current injectors and ever injectors (*r* = 41%) among all treatment demands as provided by the treatment demand indicator [29].

## Conclusion

As the latest estimates of the prevalence of ever-IDUs for Belgium date from 1995 [13], obtaining new estimates was prioritized. To be able to estimate the ever-IDU population size adopting the BM approach using the Belgian HIV/AIDS register as benchmark, statistical corrections were required. In particular, not accounting for the mortality among HIV^+^/AIDS^–^ cases would nowadays results in seriously biased estimates. By developing the improved methodology, recent ever-IDU population estimates for Belgium could be obtained. These estimates are essential to monitor trends over time and to forecast health care needs and costs (e.g. for Hepatitis C treatment).

## Competing interests

The authors declared that they have no competing interests.

## Authors’ contributions

KB conceived and carried out the statistical analysis and drafted the paper. AS provided substantial intellectual input regarding the use of the HIV/AIDS register and critically revised the manuscript. MA provided substantial intellectual input regarding the statistical analysis and critically revised the manuscript. All authors read and approved the final manuscript.

## Authors’ information

KB holds a master degree in psychology and a PhD in statistics on quantitative epidemiology. She is currently working as senior biostatistician within the research group Substance Use and Related Disorders. AS is a medical doctor and head of the HIV/AIDS register. MA is professor in biostatistics with strong interest in quantitative epidemiology.
